# Emerging Biomarkers and Targeted Therapies in Feline Mammary Carcinoma

**DOI:** 10.3390/vetsci8080164

**Published:** 2021-08-11

**Authors:** Andreia Gameiro, Ana Catarina Urbano, Fernando Ferreira

**Affiliations:** CIISA—Centro de Investigação Interdisciplinar em Sanidade Animal, Faculdade de Medicina Veterinária, Universidade de Lisboa, Avenida da Universidade Técnica, 1300-477 Lisboa, Portugal; agameiro@fmv.ulisboa.pt (A.G.); acurbano@fmv.ulisboa.pt (A.C.U.)

**Keywords:** feline mammary carcinoma, biomarkers, feline *her2* mutations, targeted therapies, comparative oncology model

## Abstract

Feline mammary carcinoma (FMC) is a common aggressive malignancy with a low survival rate that lacks viable therapeutic options beyond mastectomy. Recently, increasing efforts have been made to understand the molecular mechanisms underlying FMC development, using the knowledge gained from studies on human breast cancer to discover new diagnostic and prognostic biomarkers, thus reinforcing the utility of the cat as a cancer model. In this article, we review the current knowledge on FMC pathogenesis, biomarkers, and prognosis factors and offer new insights into novel therapeutic options for HER2-positive and triple-negative FMC subtypes.

## 1. Introduction

Cats are the most popular companion animals in developed countries, outnumbering dogs [1]. As they share similar environmental conditions with their owners, as well as genetic and biological features, cats have been used as models for human ophthalmic diseases, type 2 diabetes and, since the full sequencing of their genome, comparative oncology studies [2,3,4,5]. Cats are also emerging as promising animal models for preclinical testing of HER2-positive and triple-negative mammary carcinoma therapies [6,7,8,9,10,11]. Feline mammary carcinoma (FMC) is the third most common type of cancer in cats, corresponding to 17% of all tumors in queens, and is usually malignant [12], as is human breast cancer (HBC) [13], occurring in 90% of the cases due to somatic mutations [14] and showing comparable risk factors. It is the first cause of death in cats, with short overall survival (OS), and very poor prognosis, as it tends to be diagnosed at late stages and has limited therapeutic options that show weak responses [4,15]. FMC has similar anatomical, biological and clinical features to HBC, although metastatic mechanisms remain poorly understood [4], and is likewise classified in different molecular subtypes: luminal A, luminal B, epidermal growth factor receptor 2-positive (HER2-positive) and triple-negative normal-like and basal-like [16,17].

Using the extensive knowledge available on HBC, it is possible to find comparable diagnostic and prognostic biomarkers, as well as therapeutic targets, like the HER2 protein, that may improve FMC’s prognosis. These epidermal growth factor receptor (EGFR) family members are commonly targeted in breast cancer therapies by antibodies and/or small inhibitors that disrupt different cellular pathways [18,19,20,21,22,23,24]. Other emerging agents that have already proved valuable in FMC in vitro studies [9] include histone deacetylase inhibitors (HDACi) [25,26], and microtubules inhibitors (MTi) [27,28,29]. 

This review summarizes the similarities between FMC and HBC, with special emphasis on the progress attained in FMC, in particular towards better understanding of its clinical hallmarks and molecular and biological features. Furthermore, the antiproliferative effects of several compounds already approved for HBC therapy are discussed in the context of FMC cell-based models as future treatments proposed for cats with mammary carcinoma.

## 2. Feline Mammary Carcinoma

**Figure 1:**
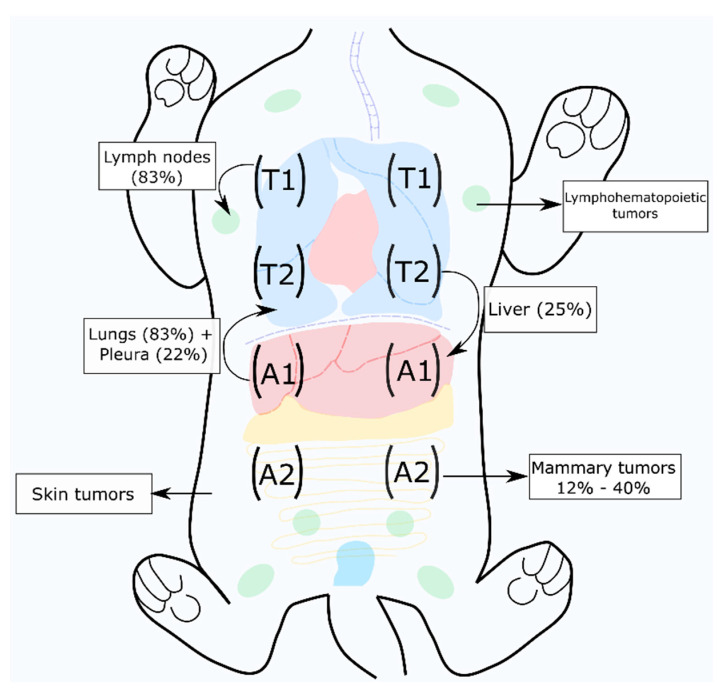
Mammary carcinoma is the third most common tumor in cats, with a high metastasis rate, frequently to lymph nodes and lungs [17,31]. The black arrows indicate the most frequent tumor locations and metastasis pattern.

FMC is a common disease in middle-aged to old queens (10 to 12 years) [30,31], more frequent in the Siamese and domestic short-hair breeds, with an OS time of around 1 year [16,17,31,32]. It occurs more frequently in unspayed cats, being associated with the expression of estrogens (ER) and progesterone (PR), and hormonal therapy [33]. Indeed, an ovariohysterectomy before six months of age is known to be a protective factor, reducing FMC development in 91% of cases [12,33]. Mammary tumors are usually malignant (80 to 90%), occurring with higher frequency in the abdominal glands and in 50% to 90% of the cases leading to metastasis [31], most commonly in the regional lymph nodes and lungs [12] (Figure 1).

At the time of diagnosis, identification of multiple masses is common, usually in the same mammary chain, whereas in women, a single mass is observed in most cases. The same anatomic classification (in situ vs. infiltrative) and histologic grade [4] are reported for FMC and HBC. Thus, mammary tumors are defined as simple or complex, with secretory and ductal cells documented, and identified as inflammatory disease, when less differentiated cells and lymphatic-dermic obstruction are present.

### 2.1. Mammary Tumor Diagnosis and Classification

Early-stage mammary tumors present as mobile, palpable, discrete masses. However, as tumor diagnosis is usually belated, patients tend to present several masses, with ulceration (25% of the cases) and necrosis. The physical exam may also reveal edema, exudate in the nipples, and a decrease in the temperature of the pelvic region. For correct diagnosis and prognosis before surgery, a precise tumor classification is mandatory. Even though cytology is easy to perform, most of the time, results are inconclusive [12], making biopsy crucial to confirm tumor stage and malignancy grade [31].

Although a standardized classification system does not exist, the same parameters used for HBC are applied (Table 1), with TNM (Tumor, Nodes and Metastasis) being the most widely used staging system [34,35]. Tumor classification also considers the malignancy grade, which takes into account tumor size, tissue invasion, ulceration, lymphovascular invasion, and lymph node status [35]. Additionally, a histopathological analysis is advised, with higher frequencies of adenocarcinomas in situ, tubullopapilary, solid, or cribiform masses reported. Concerning histologic grade, the Elston and Ellis (EE) Grading System is usually employed, with the majority of tumors defined as moderate to less differentiated masses [31,36]. Moreover, the molecular characterization in luminal A, luminal B, HER2-positive and triple-negative subtypes [17], as in women, reveals itself as an important prognostic factor, and may unveil targets for a directed therapy.

### 2.2. Feline her2 Mutations Could Be Associated with Tumor Development

Chromosomic instability is a key factor for tumor development. Concerning the HER2-positive subtype, which presents similar clinicopathological features to HBC [17] and is one of the most common in the cat (33% to 60% of all cases) [17,37], a deep analysis of the *her2* gene, by comparison to the human counterpart may be considered, as they share a 90% to 95% homology [4,38]. 

HER2 is a glycoprotein that contributes to cell proliferation, differentiation, and survival [39,40]. Interestingly, in women, breast cancer progression may be associated with HER2 amplification, conditioned by a gain in *her2* gene copy numbers, observed by in situ hybridization [41,42,43]. By comparison, a different process occurs in the cat, with an increase in *her2* mRNA copy numbers, evaluated by real-time reverse transcriptase (RT)-qPCR [44,45,46].

We know that in breast cancer patients, *her2* is mutated in 2 to 3% of primary tumors, the most common mutations occurring in the HER2-negative breast cancer subtype, which has a reported rate of incidence of 70% in the cat [10,47]. In these species, 90% of the breast tumors also have acquired somatic mutations [14], mostly occurring in the TK domain [10,11,14,46], with two single variants (SV) and two haplotypes described [38]. The observed *her2* mutations are suggestive of an association with the clinicopathological features, being correlated with primary tumor size and the number of tumor masses [10,38]. Furthermore, SVs at splicing regions, *her2* polymorphisms, or mutations in introns may be originating different isoforms of the protein, triggering the HER2 activity and tumor aggressiveness [38] or therapy resistance [48,49], as has already been described in HBC patients. Considering the *her2* gene sequence that encodes for part of the HER2 protein’s extracellular domain, three non-synonymous genomic variants were reported, predicting an alteration of the 3D structure of the protein by computational analysis and modelling [14]. 

### 2.3. Prognostic Factors for Feline Mammary Carcinoma

To uncover diagnostic and prognostic biomarkers, as well as new therapeutic targets in cats, the study of the tumor microenvironment, its molecular characterization, and the analysis of systemic alterations is crucial. 

In a macroscopic analysis, tumor size is one of the most important prognostic factors in FMC, with masses larger than 3 cm presenting a poor prognosis [36], and conditioning a more aggressive surgical approach [50]. Furthermore, the tumor’s histologic grade, presence of lymphatic metastasis and/or lymphovascular invasion [12], as well as tumor stage [51] and subtype [36], have shown to be highly correlated with OS time. 

Despite this being a relatively recent field of study, several biomarkers have already been identified that may be involved in FMC prognosis. Molecular expression of Ki-67, evaluated by immunohistochemistry [12], reveals that an index above 14% is associated with poor prognosis [52]. AKT expression, which is usually associated with PR-/ER-negative invasive carcinomas, also correlates with malignancy and non-tumor differentiation, lowering the disease-free survival (DFS) ratio [53]. In parallel, cats with a triple-negative subtype present a higher mTOR expression, as has been described in women [54], this being associated with cancer invasion and metastasis [55]. Moreover, mutations in the *p53* gene involved in cell cycle regulation and tumor suppression have been reported in 18.9% of FMCs [56,57]. Furthermore, overexpression of several molecular biomarkers is also associated with poor prognosis, e.g., macrophage-stimulating protein receptor (RON), related to tumor invasion, cyclo-oxygenase (COX)-2, expressed in malignant FMCs, and topoisomerase IIβ binding protein 1 (TopBP1), which is similar to BRAC2 in HBC [31,58]. Interestingly, the CXCR4/CXCL12 axis, which controls cell survival, migration and proliferation, is also a key factor in feline breast cancer progression and metastasis, as reported for HBC [59,60], and it’s disruption is associated with lower OS time [60,61,62]. Additionally, CXCL12 has been reported as a blood serum marker in the cat, particularly for HER2-positive tumors [62]. Finally, analysis of the vascular endothelial growth factor (VEGF) status, shows that this molecule is overexpressed in more aggressive carcinomas [50,63], playing an important role in tumor-associated angiogenesis. 

**Figure 2:**
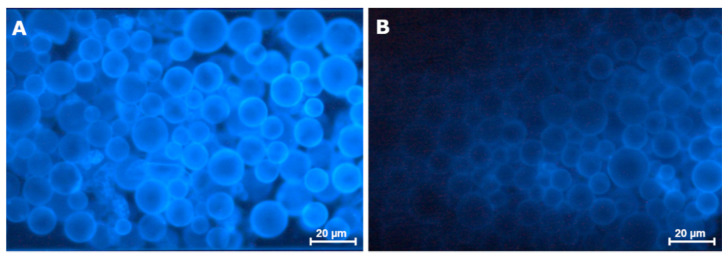
Serum HER2 protein levels measured using nanoparticles coated with an anti-HER2 antibody. (**A**) The nanoparticles were coated with a fluorescent anti-HER2 antibody (CB11), allowing quantification of serum HER2 expression levels in cats with HER2-positive mammary carcinoma (3+ score), by comparison with a (**B**) control serum sample from a healthy animal. This experiment corresponds to preliminary results from a recent study (data not published; 400× magnification).

In parallel, an association is reported between the expression of some of these proteins in serum and in the tumor microenvironment, suggesting that serum samples may be used as a non-invasive method for the assessment of checkpoint molecules [64,65,66]. Interestingly, in the cat, HER2 serum expression is elevated in malignant lesions, lowering OS [17,31], a fact that makes it a promising diagnostic tool. In fact, our group has already shown that a rapid diagnostic kit for the identification of HER2-positive mammary carcinoma through detection of serum HER2 expression levels can be produced. These preliminary experiments, using nanoparticles coated with anti-HER2 fluorescent antibodies, showed that serum HER2 expression levels can be quantified in cats with mammary carcinoma (Figure 2A), by comparison with a control sample (Figure 2B; data not published). Nevertheless, more work is needed in order to define cut-off values, sensitivity and specificity of the test.

It’s widely acknowledged that in women, a chronic inflammatory status, such as that induced by obesity, can be a trigger for mammary tumor development [67,68]. Interestingly, cats with mammary carcinoma present a decrease in the serum leptin/leptin receptor (ObR) ratio [66,69], as has been documented for pre-menopausal women with breast cancer [70]. Furthermore, in animals with FMC, the higher leptin levels are associated with a triple-negative tumor subtype, also as reported for HBC [66,71,72]. In parallel, ObR is associated with an immunosuppressive status [66,73,74], observed in both breast cancer patients [75,76] and cats with mammary carcinoma, and is additionally correlated with the overexpression of cytotoxic T-lymphocyte associated protein 4 (CTLA-4), tumor necrosis factor-α (TNF-α) [64,77], and programmed cell death (PD-1)/programmed death ligand-1 (PD-L1) [64,78] in the most aggressive tumor subtypes (HER2-positive and triple-negative) [64,79].

## 3. Feline Mammary Carcinoma Cell-Based Models for Targeted Therapies

In cats, therapeutic options are scarce, the most common being uni/bilateral radical mastectomy, alone, or in combination with chemotherapeutic adjuvant protocols when the Ki-67 index is above 14% [52], which increases the cat’s DFS but not OS, due to the high metastasis rate [12,31,80]. Moreover, the agents used tend to have limited efficacy and severe side effects [4,15]. Combination therapy protocols with doxorubicin and cyclophosphamide/carboplatin, for example, show poor response in metastasis [31,81], and tamoxifen shows no significant response [4], as FMC is more commonly ER-negative, unlike HBC [4,33]. 

**Figure 3:**
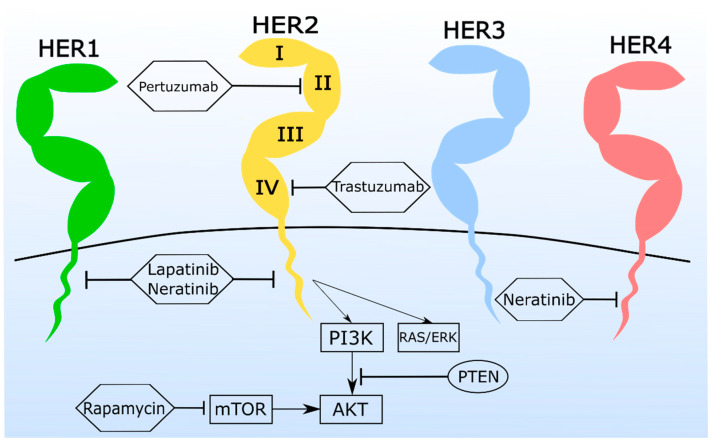
The HER2 pathway is a common target in human breast cancer therapy, revealing promising results for the treatment of cats with mammary tumors.

Thus, a deep understanding is needed to unveil alternative therapeutic options aimed at improving the cat’s clinical outcome. Such studies are limited, however, by a lack of feline cell lines available for cytotoxicity assays, with only 8 having been reported so far [82] and a shortage of in vivo models for preclinical trials, although four FMC xenograft models were recently reported (preliminary report) [83], revealing to be of extreme importance in order to understand the mammary carcinoma biology, development and metastization process [84,85], as the use of nude mouse models [84]. Here we review some therapeutic drugs (Figure 3) approved for HBC therapy that were recently tested in FMC cell-based models (Table 2; CAT-MT from the European Collection of Authenticated Cell Culture, England; FMCp and FMCm kindly provided by Prof. Nobuo Sasaki and Prof. Takayuki Nakagawa, University of Tokyo, Japan). The reported results represent an initial step towards the development of more effective therapeutic options for cats with mammary carcinoma and interestingly, all assays reveal promising results and a conserved mechanism of action [10,11], by comparison to a human HER2-overexpressing cell line (SKBR-3; American Type Culture Collection [86]).

More in vitro studies are needed, however, to fully characterize the effect of the antitumor compounds tested, as well as develop proper xenograft models for preclinical studies.

Furthermore, despite the real value of FMC xenograft models, some limitations could be pointed out, such as the need of induced tumors, absence of a competent immune system, or comparable pharmacokinetic and pharmacodynamics responses, when compared to other mammals [87]. Thus, the use of the cat as an in vivo oncology model for HBC reveal several advantages to take into consideration, representing epidemiologic, clinical and morphologic similarities with its human counterpart [88].

### 3.1. Monoclonal Antibodies (mAbs) and Antibody-Drug Conjugates (ADC) Are a Promising Tool for the Treatment of Feline Mammary Carcinoma

The HER2 protein is a common target for molecular therapy in HBC patients, using mAbs that interact by shape complementarity [18], thus preventing HER2 dimerization and activation of its downstream pathways [89]. These compounds are a good alternative to Tyrosine Kinase inhibitors (TKi), which are toxic for the majority of tissues, showing severe side effects [90]. Recent studies have revealed a 93% similarity between human and feline HER2 [6,14] (*homo sapiens*, UniProt P04626; and *felis catus,* UniProt H9BB15), which allowed for testing of humanized-mAbs against FMC (Table 3). 

In the assays testing both mAbs (pertuzumab and trastuzumab), a dose-dependent antiproliferative effect, as well as a conserved cell death mechanism by apoptosis, were demonstrated, even though feline cell lines present lower HER2 expression levels when compared to the human SkBR-3 cell line [11].

In the pertuzumab assay, the addition of heregulin to the cell medium was suggested, which would allow for mAb-HER2 heterodimerization [104,105], thus improving cytotoxicity results. Furthermore, an antiproliferative effect was described in the FMCp HER2-negative cell line [11,53,55]. In fact, pertuzumab has already been suggested for the treatment of triple-negative HBC expressing HER2-103, a recently described protein encoded by a circular form of the HER2 gene that is associated with worse overall prognosis for these patients [106]. A pertuzumab-HER3 interaction has also been reported in human lung cancer [107]. This suggests there may be a real benefit of pertuzumab in the treatment of HER2-negative FMC. However, more studies are needed. In parallel, testing of trastuzumab on the same FMCp cell line also revealed a promising antiproliferative effect. Despite the lower cytotoxic response, due to a lack of HER2 expression, this result proposes that cats with HER2-negative tumors may benefit from the use of trastuzumab, as suggested for human triple-negative breast cancer that expresses an activated form of HER2 (HER2^Y877^) [108].

Other compounds used for the treatment of breast cancer are the ADCs, e.g., trastuzumab-emtansine (T-DM1). This ADC allows a targeted delivery of the cytotoxic agent, DM-1 a microtubule inhibitor, to HER2-overexpressing tumor cells, decreasing its side effects [101]. Testing of T-DM1 in FMC cell-based models resulted in promising cytotoxic effects, leading to a conserved cell death mechanism by apoptosis. Interestingly, for the HER2-negative FMCp cell line a high cytotoxic effect was observed [11], which could be explained by the interaction of DM1 with the cytoskeleton-associated protein 5 (CKAP5), a microtubule assembly regulator, as described in human HER2-negative cells [109]. More studies are needed to evaluate the expression status of the CKAP5 protein in triple-negative FMC and be in a better position to propose T-DM1 for the treatment of feline HER2-negative breast cancer.

Despite the good results of these cytotoxicity assays, a 3D cell culture system is needed for correct prediction of receptor-mAb conformational interactions [110,111], and proper felinized mAbs should be designed.

### 3.2. Tyrosine Kinase Inhibitors (TKi) Are Valuable in Feline Mammary Carcinoma Therapy

TKis are small chemical compounds that prevent protein phosphorylation, by interacting with the cytoplasmic catalytic kinase domain [21], for example, of EGFR family members. These compounds block HER2 signaling for cell proliferation via the RAS-ERK pathway [112] and for cell death inhibition via the PI3K-AKT-mTOR pathway [113]. Despite their side effects, they are a suitable alternative for patients that show resistance to anti-HER2 mAbs, which in women with HER2-positive breast cancer is around 50% [21,114]. TKis (lapatinb and neratinib) have been tested against FMC in in vitro models, with promising cytotoxic effects obtained (Table 4).

The lapatinib exposure assay demonstrated a dose-dependent antiproliferative effect with a conserved mechanism of action, by reducing HER1 (Y1173) and HER2 (Y1221+Y1222) phosphorylation patterns, and their downstream pathways, AKT (S473) and ERK 1/2 (T202/Y204+T185/Y187), involved in cell cycle progression and apoptosis [10,120,121]. Interestingly, like in the feline HER2-positive cell lines tested (CAT-M and FMCm), the feline HER2-negative cell line (FMCp) presented a 100% cytotoxic response [10]. These results suggest an interaction between lapatinib and HER1, which is an EGFR family member usually upregulated in women with triple-negative breast tumors [122,123]. Moreover, lapatinib is described as activating NF-kB in triple-negative HBC, inducing cell apoptosis [124,125], a different pathway that should be investigated in cats. This study also showed that lapatinib induces the accumulation of membrane HER2 [10,125], suggesting protein stabilization by the inhibition of HER2 phosphorylation and prevention of receptor ubiquitination [126], as described for human cells.

In parallel, neratinib assay revealed similar antiproliferative effects in all feline cell lines tested, including the FMCp HER2-negative cell line [10], which may suggest an interaction with other EGFR family members, such as HER1 [122,123], or HER4 [127,128]. In contrast, a dose-dependent effect was not observed in the FMCm metastatic cell line [10], suggesting a resistance pattern, as has been documented in humans, e.g., because of increased activity of the cytochrome P4503A4 [129], or overexpression of NmU, a protein involved in breast cancer progression and metastasis [130].

At this point, the need for an in vivo system arises to characterize the cats’ systemic response to these compounds.

### 3.3. Combination Therapy Shows Synergistic Antiproliferative Effects in Feline Mammary Carcinoma Cell Lines

Acquired resistance to therapy is a well-documented phenomena in women, and in order to surpass this and improve patients’ clinical outcome, combined therapies have become a valuable tool [131]. Different combinations are found in the literature, e.g., of different mAbs [22,131,132], of mAbs with TKis [133,134,135], and of TKis with the mTOR inhibitor (mTORi) rapamycin [136,137]. In this way, we are able to block different cell proliferation and survival signaling pathways [6,32,40,54] using lower drug concentrations (Table 5), and this is a strategy that could become important in cats.

It is known that pertuzumab is complementary to trastuzumab in HBC mAb combined therapy [92], presenting a synergistic antiproliferative effect, and this same effect has been observed in FMC cell lines [11].

Combination therapy with the mAb pertuzumab and the TKi, lapatinib, also shows a synergistic effect in FMC cell-based models. This effect is particularly noticeable in the FMCp cell line [11], as the combined drugs are able to target different EGFR family members [106]. This combination was effective in the metastatic FMCm cell line as well [11], a promising result, as it has already been approved in humans for metastatic tumors [115,116]. Trastuzumab also acts synergistically with lapatinib revealing an additive antiproliferative effect in FMC cell lines [11]. In fact, this protocol has already proved effective against HBC, particularly for HER2-positive and metastatic therapy, improving patients DFS [126,138]. 

In conjugation protocols between TKis and rapamycin it is important to characterize mTORi effects. The mTOR pathway [139,140] is the target of rapamycin in adjuvant protocols. This compound presents immunosuppressant anticancer properties, but with no effective cytotoxic response when used as a single agent [10], as described for human cancers [54]. Interestingly, in the FMCp HER2-negative cell line, good results were obtained, which could be explained by mTOR overexpression, something that has been reported in cats with HER2-negative mammary carcinomas [55] and also breast cancer patients [141]. Its conjugation with lapatinib, however, reveals a synergistic antiproliferative response in all feline cell lines [10]. Thus, it may be described as a valuable tool in combined protocols, namely for metastatic breast cancer therapy [10,115,116]. In parallel, conjugation with neratinib also reveals synergistic antiproliferative effects, particularly noticeable in the FMCm and FMCp cell lines [10], being this protocol recommended for human metastatic HER2-positive and triple-negative breast cancer therapy [142,143].

### 3.4. Novel In Vitro Approaches to Feline Mammary Carcinoma Therapy

Current knowledge on HBC reveals different tumor subtypes, e.g., the triple-negative, which has no directed therapy [55], as well as development of therapeutic resistance, which requires different strategies to improve patients clinical outcome. Highlighting the importance of the cat as a model, and since few studies exist [144,145], the antiproliferative effects of new compounds, e.g., HDACi [146] and MTi [147], were recently tested in FMC in vitro models (CAT-M and FMCp), revealing themselves as promising agents for molecular targeted therapy (Table 6).

Histone deacetylases are enzymes that control gene expression, and their dysregulation is associated with tumor development [150,151]. Thus, in the past few years, they have been investigated as potential antitumor agents. In parallel, microtubules are tubulin polymers essentials for cell growth, division and intracellular trafficking [28], and are known to be valuable targets for tumor therapy in women. Interestingly, several HDACis (CI-994, panabinostat, SAHA, SBHA, scriptaid and trichostatin A) and MTis (colchicine, nocodazole, paclitaxel and vinblastine) that have been tested in FMC cell lines show a dose-dependent antiproliferative effect and conserved cell death mechanism, by apoptosis. Furthermore, using HDACi it was possible to demonstrate an accumulation of the acetylated form of the histone H3 (Lys9/Lys14), as described in humans [9,152].

## 4. Conclusions

The cat is considered a good oncology model [4], namely for HER2-positive and triple-negative breast cancers [17,55], although more efforts are needed to better understand the development mechanism and biology of FMC.

FMC tends to be diagnosed belatedly, presenting ulcerated masses, or metastasis [31], and the therapeutic alternatives available are scarce, being restricted to mastectomy [80] and adjuvant therapeutic protocols, with, however, limited success [4,31,50]. With this in mind, research groups are now directing their attention to the in vitro study of drugs already approved for HBC therapy on FMC cell-based models, demonstrating promising antiproliferative effects of several compounds. Furthermore, through the analysis of mammary carcinoma clinical samples, it has been possible to show that the cat does not present any known mutations thought to lead to resistance to therapy [10,11]. Moreover, similarities between the feline and human tumor micro- and serological environments have also been revealed, suggesting equivalent tumor diagnostic and prognostic biomarkers, as well as the possible use of adjuvant treatments recommended in breast cancer therapeutic protocols [64,66,153,154]. This introduces a new research line, e.g., the use of anti-leptin [153], anti-PD1 [154], or anti-VEGF [155] molecules.

Forthcoming perspectives include a deeper knowledge of FMC, defining proper diagnostic and prognostic biomarkers that can be used in clinical practice, and improvement of therapeutic options for cats. Additionally, we point out that, in the near future, a fast diagnostic kit to identify serum protein expression levels in cats with mammary carcinomas may become available, e.g., in the HER2-positive subtype, one of the most aggressive tumors [17], which would allow prediction of prognosis and inform the choice of therapeutic protocol.

## Figures and Tables

**Table 1:** Tumor clinical stage and histological grade for feline mammary carcinoma. (Adapted from the System modified from Owen LN., Classification of tumors in domestic animals, Geneva World Health Organization, 1980; and Elston & Ellis Grading System, 1998, respectively).

Tumor Classification of Feline Mammary Carcinomas			
Tumor Clinical Stage *			
Stage	Tumor size (T)	Lymph node status (N)	Metastasis (M)
1	T1 (<2 cm)	N0	M0
2	T2 (2–3 cm)	N0	M0
	T1	N1	M0
3	T2	N1	M0
	T3 (>3 cm)	N0/N1	M0
4	Any	N0/N1	M1
**Histological Grade (EE System)**			
Histologic feature	Score	**Sum of the scores** 3–5 6–7 8–9	**Grade** I II III
Tubule formation			
>75%	1		
10–75%	2		
<10%	3		
Nuclear pleomorphism			
Mild	1		
Moderate	2		
Marked	3		
Mitotic count (per 10 microscopic fields)			
0–5	1		
6–10	2		
>11	3		

* 0—indicates absence of the characteristic; 1—indicates presence of the characteristic.

**Table 2:** Classification and molecular characterization of FMC cell lines [10,11].

Cell Line	Tumor Classification	ER (%)	PR (%)	Ki-67 (%)	Ck5/6 (%)	HER2
CAT-M	Mammary Adenocarcinoma	10	80	50.2	<1	2+
FMCp	Primary breast tumor	60	Negative	57.4	<1	0
FMCm	Metastatic lymph node	2	Negative	68.5	<1	1+

ER—Estrogen Receptor; PR—Progesterone Receptor; Ck5/6 and HER2 [17]; Ki-67 index [52].

**Table 3:** Monoclonal antibodies (pertuzumab and trastuzumab) and antibody-drug conjugate (T-DM1) compound induce cell apoptosis, with promising antiproliferative effects in FMC in vitro models [11].

mAb	Target	Mechanism of Action	Breast Cancer Clinical Application	References	FMC In Vitro System			
					Cell Line	HER2 Status	Concentration (µg/mL)	Cytotoxicity (%)
Pertuzumab	HER2 ECD II	Prevents HER2 heterodimerization; Inhibits EGFR downstream pathways; Stimulates ADCC and apoptosis	HER2-overexpressing and metastatic tumors	Agus et al., 2002 [91]; Scheuer et al., 2009 [92]; Baselga et al., 2010 [93]; Metzger-Filho et al., 2013 [94]; Richard et al., 2016 [22]; and Yamashita-Kashima et al., 2017 [89]	CAT-M	2+	10,000 (EC_50_ = 2837.92 µg/mL ± 1.50)	60.2
					FMCp	0	10,000 (EC_50_ = 928.97 µg/mL ± 1.11)	52.1
					FMCm	1+	10,000 (EC_50_ = 1205.04 µg/mL ± 1.23)	61.8
Trastuzumab	HER2 ECD IV	Prevents HER2 homodimerization; Block receptor internalization and degradation; Prevents HER2 shedding; Induces ADCC and apoptosis	HER2-overexpressing invasive, metastatic and early-stage tumors	Klapper et al., 2000 [95]; Cho et al., 2003 [18]; J. Piccart-Gebhart, 2005 [96]; Nahta et al., 2007 [97]; D. Slamon, 2011 [98]; Menyhart et al., 2015 [99]; Richard et al., 2016 [22]; and Kast et al., 2017 [100]	CAT-M	2+	10,000 (EC_50_ = 3047.89 µg/mL ± 1.43)	92.6
					FMCp	0	10,000 (EC_50_ = 3243.40 µg/mL ± 2.29)	60.1
					FMCm	1+	10,000 (EC_50_ = 528.45 µg/mL ± 1.14)	82.7
T-DM1	HER2 ECD II; CKAP5	Prevents HER2 homodimerization; Inhibits microtubule assembly; Induces cell apoptosis	HER2-positive, advanced, early stage and metastatic tumors	Phillips et al., 2008 [19]; Lambert and Chari, 2014 [101]; Von Minckwitz et al., 2019 [24]; Lacasse et al., 2020 [102]; and Liu et al., 2020 [103]	CAT-M	2+	1000 (EC_50_ = 19.63 µg/mL ± 1.22)	94.0
					FMCp	0	1000 (EC_50_ = 88.72 µg/mL ± 1.29)	74.2
					FMCm	1+	1000 (EC_50_ = 52.84 µg/mL ± 1.50)	53.8

**Table 4:** Tyrosine kinase inhibitors (lapatinib and neratinb) presented valuable cytotoxic effects in the FMC in vitro models, suggesting a conserved mechanism of action [10].

TKi	Target	Mechanism of Action	Breast Cancer Clinical Application	References	FMC In Vitro System			
					Cell Line	HER2 Status	Concentration (nM)	Cytotoxicity (%)
Lapatinib	HER1 and HER2	Reversible; Prevents EGFR family members phosphorylation	Solid, advanced and metastatic HER2-positive tumors; Valuable in combined protocols	Frenel et al., 2009 [115]; Opdam et al., 2012 [116]; Shi et al., 2016 [117]; and Stanley et al., 2017 [118]	CAT-M	2+	50,000 (IC_50_ = 3930 nM ± 49)	100
					FMCp	0	50,000 (IC_50_ = 4870 nM ± 100)	100
					FMCm	1+	100 × 10^3^ (IC_50_ = 17,470 nM ± 100)	100
Neratinib	HER1; HER2 and HER4	Irreversible; Prevents EGFR family members phosphorylation; Surpass lapatinib resistance	Adjuvant treatment of HER2-positive early-stage and metastatic breast cancer	Tiwari et al., 2015 [119]; Sun et al., 2015 [40]; Cocco et al., 2018 [23]; and Food and Drug Administration (FDA)	CAT-M	2+	25	33.5
					FMCp	0	250	79.4
					FMCm	1+	1000	31.4

**Table 5:** Combined protocols present synergistic antiproliferative effects in FMC cell-based models by blocking different HER2 pathways [10,11].

Combined Protocol	Blocked Pathways	FMC In Vitro Assay			
		Cell Line	HER2 Status	Increase in Cell Cytotoxicity (%)	*p*-Value
**mAbs combination**					
Pertuzumab plus Trastuzumab	HER2 ECD II and HER2 ECD IV	CAT-M	2+	26.4	0.0018
		FMCp	0	11.7	0.0184
		FMCm	1+	29.5	<0.001
**mAb plus TKi**					
Pertuzumab plus Lapatinib	HER2 ECD II; HER1 and HER2 TK domain	CAT-M	2+	69.4	<0.001
		FMCp	0	47.5	<0.001
		FMCm	1+	41.5	<0.001
Trastuzumab plus Lapatinib	HER2 ECD IV; HER1 and HER2 TK domain	CAT-M	2+	71.9	<0.001
		FMCp	0	62.0	<0.001
		FMCm	1+	27.2	0.0017
**TKi plus mTORi**					
Lapatinib plus Rapamycin	HER1 and HER2 TK domain and mTOR complex	CAT-M	2+	51.9	0.0360
		FMCp	0	47.5	<0.001
		FMCm	1+	85.6	<0.001
Neratinib plus Rapamycin	HER1, HER2 and HER4 TK domain and mTOR complex	CAT-M	2+	47.4	0.0044
		FMCp	0	44.1	0.0034
		FMCm	1+	66.7	<0.001

**Table 6:** Histone deacetylase inhibitors and microtubule inhibitors show promising cytotoxic effects in FMC cell-based models, suggesting a conserved mechanism of action [9].

Class of the Compound	Mechanism of Action	References	Agent	FDA Approval	FMC In Vitro Assays	
					Cell Line	IC_50_ Value
HDACi (µM)	Inhibits histone deacetylases leading to chromatin relaxation and uncontrolled gene expression; Induces cell cytotoxicity and death by apoptosis	Xu et al., 2007 [148]; Chun, 2015 [149]; and FDA	CI-994	Experimental	CAT-M	16.470 ± 1.904
					FMCp	9.616 ± 2.150
			Panobinostat	Yes; 2015	CAT-M	0.042 ± 0.067
					FMCp	ND ^#^
			SAHA	Yes; 2006	CAT-M	4.416 ± 0.453
					FMCp	2.571 ± 0.578
			SBHA	Experimental	CAT-M	45.230 ± 4.692
					FMCp	33.830 ± 6.454
			Scriptaid	ND	CAT-M	3.392 ± 0.403
					FMCp	3.090 ± 0.691
			Trichostatin A	Experimental	CAT-M	0.263 ± 0.062
					FMCp	ND ^#^
MTi (nM)	Inhibits microtubule polymerization, leading to cytoskeleton disruption; Induces cell cycle arrest and apoptosis	Risinger et al., 2015 [27]; Zang et al., 2018 [29]; Steinmetz and Prota, 2018 [28]; and FDA	Colchicine (Destabilizing agent)	Yes; 2009	CAT-M	1.472 ± 0.484
					FMCp	5.876 ± 0.968
			Nocodazol (Destabilizing agent)	Experimental	CAT-M	12.270 ± 3.455
					FMCp	30.840 ± 8.499
			Vinblastine (Destabilizing agent)	Yes; 2011	CAT-M	0.570 ± 1.080
					FMCp	6.563 ± 1.514
			Paclitaxel (Stabilizing agent)	Yes; 2002	CAT-M	1.939 ± 1.134
					FMCp	8.646 ± 2.337

^#^ ND—Not determined.

## Data Availability

Data sharing not applicable.

## References

[1] Murray J.K., Gruffydd-Jones T.J., Roberts M.A., Browne W.J. (2015). Assessing changes in the UK pet cat and dog populations: Numbers and household ownership. Vet. Rec..

[2] Hoenig M., Hall G., Ferguson D., Jordan K., Henson M., Johnson K., O’Brien T. (2000). A feline model of experimentally induced islet amyloidosis. Am. J. Pathol..

[3] Henson M.S., O’Brien T.D. (2006). Feline models of type 2 diabetes mellitus. ILAR J..

[4] Zappulli V., De Zan G., Cardazzo B., Bargelloni L., Castagnaro M. (2005). Feline mammary tumours in comparative oncology. J. Dairy Res..

[5] Menotti-Raymond M., Deckman K.H., David V., Myrkalo J., O’Brien S.J., Narfström K. (2010). Mutation discovered in a feline model of human congenital retinal blinding disease. Investig. Ophthalmol. Vis. Sci..

[6] De Maria R., Olivero M., Iussich S., Nakaichi M., Murata T., Biolatti B., Di Renzo M.F., Flavia M., Renzo D., Di Renzo M.F. (2005). Spontaneous feline mammary carcinoma is a model of HER2 overexpressing poor prognosis human breast cancer. Cancer Res..

[7] Burrai G.P., Mohammed S.I., Miller M.A., Marras V., Pirino S., Addis M.F., Uzzau S., Antuofermo E. (2010). Spontaneous feline mammary intraepithelial lesions as a model for human estrogen receptor and progesterone receptor-negative breast lesions. BMC Cancer.

[8] Wiese D.A., Thaiwong T., Yuzbasiyan-Gurkan V., Kiupel M. (2013). Feline mammary basal-like adenocarcinomas: A potential model for human triple-negative breast cancer (TNBC) with basal-like subtype. BMC Cancer.

[9] Almeida F., Gameiro A., Correia J., Ferreira F. (2021). Histone Deacetylase Inhibitors and Microtubule Inhibitors Induce Apoptosis in Feline Luminal Mammary Carcinoma Cells. Animals.

[10] Gameiro A., Almeida F., Nascimento C., Correia J. (2021). Tyrosine Kinase Inhibitors Are Promising Therapeutic Tools for Cats with HER2-Positive Mammary Carcinoma. Pharmaceutics.

[11] Gameiro A., Nascimento C., Correia J., Ferreira F. (2021). HER2-Targeted Immunotherapy and Combined Protocols Showed Promising Antiproliferative Effects in Feline Mammary Carcinoma Cell-Based Models. Cancers.

[12] Novosad C.A. (2003). Principles of Treatment for Mammary Gland Tumors. Clin. Tech. Small Anim. Pract..

[13] Panieri E. (2012). Breast cancer screening in developing countries. Best Pract. Res. Clin. Obstet. Gynaecol..

[14] Santos S., Baptista C.S., Abreu R.M.V., Bastos E., Amorim I., Gut I.G., Gärtner F., Chaves R. (2013). ERBB2 in cat mammary neoplasias disclosed a positive correlation between RNA and protein low expression levels: A model for erbB-2 negative human breast cancer. PLoS ONE.

[15] Vail D.M., Macewen E.G. (2000). Spontaneously occurring tumors of companion animals as models for human cancer. Cancer Investig..

[16] Soares M., Correia J., Peleteiro M.C., Ferreira F. (2016). St Gallen molecular subtypes in feline mammary carcinoma and paired metastases—disease progression and clinical implications from a 3-year follow-up study. Tumor Biol..

[17] Soares M., Madeira S., Correira J., Peleteiro M., Cardoso F., Ferreira F., Correia J., Peleteiro M., Cardoso F., Ferreira F. (2016). Molecular based subtyping of feline mammary carcinomas and clinicopathological characterization. Breast.

[18] Cho H.-S., Mason K., Ramyar K.X., Stanley A.M., Gabelli S.B., Denney D.W., Leahy D.J. (2003). Structure of the extracellular region of HER2 alone and in complex with the Herceptin Fab. Nature.

[19] Phillips G.D.L., Li G., Dugger D.L., Crocker L.M., Parsons K.L., Mai E., Blättler W.A., Lambert J.M., Chari R.V.J., Lutz R.J. (2008). Targeting HER2-positive breast cancer with trastuzumab-DM1, an antibody-cytotoxic drug conjugate. Cancer Res..

[20] Canonici A., Gijsen M., Mullooly M., Bennett R., Bouguern N., Pedersen K., O’Brien N.A., Roxanis I., Li J.-L., Bridge E. (2013). Neratinib overcomes trastuzumab resistance in HER2 amplified breast cancer. Oncotarget.

[21] Schroeder R.L., Stevens C.L., Sridhar J. (2014). Small molecule tyrosine kinase inhibitors of ErbB2/HER2/Neu in the treatment of aggressive breast cancer. Molecules.

[22] Richard S., Selle F., Lotz J.P., Khalil A., Gligorov J., Grazziotin-Soares D. (2016). Pertuzumab and trastuzumab: The rationale way to synergy. An. Acad. Bras. Cienc..

[23] Cocco E., Carmona F.J., Razavi P., Won H.H., Cai Y., Rossi V., Chan C., Cownie J., Soong J., Toska E. (2018). Neratinib is effective in breast tumors bearing both amplification and mutation of ERBB2 ( HER2 ). Sci. Signal..

[24] von Minckwitz G., Huang C.-S.S., Mano M.S., Loibl S., Mamounas E.P., Untch M., Wolmark N., Rastogi P., Schneeweiss A., Redondo A. (2019). Trastuzumab emtansine for residual invasive HER2-positive breast cancer. N. Engl. J. Med..

[25] Munster P.N., Troso-Sandoval T., Rosen N., Rifkind R., Marks P.A., Richon V.M. (2001). The histone deacetylase inhibitor suberoylanilide hydroxamic acid induces differentiation of human breast cancer cells. Cancer Res..

[26] Huang L., Pardee A.B. (2000). Suberoylanilide hydroxamic acid as a potential therapeutic agent for human breast cancer treatment. Mol. Med..

[27] Risinger A.L., Dybdal-Hargreaves N.F., Mooberry S.L. (2015). Breast cancer cell lines exhibit differential sensitivities to microtubule-targeting drugs independent of doubling time. Anticancer. Res..

[28] Steinmetz M.O., Prota A.E. (2018). Microtubule-Targeting Agents: Strategies To Hijack the Cytoskeleton. Trends Cell Biol..

[29] Zang X., Wang G., Cai Q., Zheng X., Zhang J., Chen Q., Wu B., Zhu X., Hao H., Zhou F. (2018). A promising microtubule inhibitor deoxypodophyllotoxin exhibits better efficacy to multidrug-resistant breast cancer than paclitaxel via avoiding efflux transport. Drug Metab. Dispos..

[30] Weijer K., Head K.W., Misdorp W., Hampe J.F. (1972). Feline malignant mammary tumors. I. morphology and biology: Some comparisons with human and canine mammary carcinomas1, 2. J. Natl. Cancer Inst..

[31] Giménez F., Hecht S., Craig L.E., Legendre A.M. (2010). Early Detection, Aggressive Therapy. J. Feline Med. Surg..

[32] Millanta F., Calandrella M., Citi S., della Santa D., Poli A. (2005). Overexpression of HER-2 in feline invasive mammary carcinomas: An immunohistochemical survey and evaluation of its prognostic potential. Vet. Pathol..

[33] Overley B., Shofer F.S., Goldschmidt M.H., Sherer D., Sorenmo K.U. (2005). Association between ovarihysterectomy and feline mammary carcinoma. J. Vet. Intern. Med..

[34] Preziosi R., Sarli G., Benazzi C., Mandrioli L., Marcato P.S. (2002). Multiparametric survival analysis of histological stage and proliferative activity in feline mammary carcinomas. Res. Vet. Sci..

[35] Chocteau F., Boulay M.M., Besnard F., Valeau G., Loussouarn D., Nguyen F. (2019). Proposal for a Histological Staging System of Mammary Carcinomas in Dogs and Cats. Part 2: Feline Mammary Carcinomas. Front. Vet. Sci..

[36] Mills S.W., Musil K.M., Davies J.L., Hendrick S., Duncan C., Jackson M.L., Kidney B., Philibert H., Wobeser B.K., Simko E. (2015). Prognostic Value of Histologic Grading for Feline Mammary Carcinoma: A Retrospective Survival Analysis. Vet. Pathol..

[37] Soares M., Ribeiro R., Najmudin S., Gameiro A., Rodrigues R., Cardoso F., Ferreira F. (2016). Serum HER2 levels are increased in cats with mammary carcinomas and predict tissue HER2 status. Oncotarget.

[38] Santos S., Bastos E., Baptista C.S., Sá D., Caloustian C., Guedes-Pinto H., Gärtner F., Gut I.G., Chaves R. (2012). Sequence variants and haplotype analysis of cat ERBB2 gene: A survey on spontaneous cat mammary neoplastic and non-neoplastic lesions. Int. J. Mol. Sci..

[39] Witton C.J., Reeves J.R., Going J.J., Cooke T.G., Barlett J.M.S. (2003). Expression of the HER1-4 family of receptor tyrosine kinases in breast cancer. J. Pathol..

[40] Sun Y., Feng X., Qu J., Han W., Liu Z., Li X., Zou M., Zhen Y., Zhu J. (2015). Expression and Characterization of the Extracellular Domain of Human HER2 from Escherichia Coli, and Production of Polyclonal Antibodies Against the Recombinant Proteins. Appl. Biochem. Biotechnol..

[41] Meng S., Tripathy D., Shete S., Ashfaq R., Haley B., Perkins S., Beitsch P., Khan A., Euhus D., Osborne C. (2004). HER-2 gene amplification can be acquired as breast cancer progresses. Proc. Natl. Acad. Sci. USA.

[42] Jiang H., Bai X., Zhang C., Zhang X. (2012). Evaluation of HER2 gene amplification in breast cancer using nuclei microarray in Situ hybridization. Int. J. Mol. Sci..

[43] Vicario R., Peg V., Morancho B., Zacarias-Fluck M., Zhang J., Martínez-Barriocanal Á., Jiménez A.N., Aura C., Burgues O., Lluch A. (2015). Patterns of HER2 gene amplification and response to anti-HER2 therapies. PLoS ONE.

[44] Soares M., Correia J., Rodrigues P., Simões M., de Matos A., Ferreira F. (2013). Feline HER2 Protein Expression Levels and Gene Status in Feline Mammary Carcinoma: Optimization of Immunohistochemistry (IHC) and In Situ Hybridization (ISH) Techniques. Microsc. Microanal..

[45] Ferreira D., Soares M., Correia J., Adega F., Ferreira F., Chaves R. (2019). Assessment of ERBB2 and TOP2agene statusand expression profilein feline mammary tumors: Findings and guidelines. Aging.

[46] Muscatello L.V., Di Oto E., Sarli G., Monti V., Foschini M.P., Benazzi C., Brunetti B. (2019). HER2 Amplification Status in Feline Mammary Carcinoma: A Tissue Microarray–Fluorescence In Situ Hydridization–Based Study. Vet. Pathol..

[47] Hanker A.B., Brewer M.R., Sheehan J.H., Koch J.P., Sliwoski G.R., Nagy R., Lanman R., Berger M.F., Hyman D.M., Solit D.B. (2017). An acquired HER2T798Igatekeeper mutation induces resistance to neratinib in a patient with HER2 mutant-driven breast cancer. Cancer Discov..

[48] Rockberg J., Schwenk J.M., Uhlén M. (2009). Discovery of epitopes for targeting the human epidermal growth factor receptor 2 (HER2) with antibodies. Mol. Oncol..

[49] Kanthala S., Mill C.P., Riese D.J., Jaiswal M., Jois S. (2016). Expression and purification of HER2 extracellular domain proteins in Schneider2 insect cells. Protein Expr. Purif..

[50] McNeill C.J., Sorenmo K.U., Shofer F.S., Gibeon L., Durham A.C., Barber L.G., Baez J.L., Overley B. (2009). Evaluation of Adjuvant Doxorubicin-Based Chemotherapy for the Treatment of Feline Mammary Carcinoma. J. Chem. Inf. Model..

[51] Ito T., Kadosawa T., Mochizuki M., Matsunaga S., Nishimura R., Sasaki N. (1996). Prognosis of malignant mammary tumor in 53 cats. J. Vet. Med. Sci..

[52] Soares M., Ribeiro R., Carvalho S., Peleteiro M., Correia J., Ferreira F. (2016). Ki-67 as a Prognostic Factor in Feline Mammary Carcinoma: What Is the Optimal Cutoff Value?. Vet. Pathol..

[53] Maniscalco L., Iussich S., de Las Mulas J.M., Millán Y., Biolatti B., Sasaki N., Nakagawa T., De Maria R., Martín de las Mulas J., Millán Y. (2012). Activation of AKT in feline mammary carcinoma: A new prognostic factor for feline mammary tumours. Vet. J..

[54] Watanabe R., Wei L., Huang J. (2011). mTOR Signaling, Function, Novel Inhibitors, and Therapeutic Targets. J. Nucl. Med..

[55] Maniscalco L., Millan Y., Iussich S., Denina M., Sanchez-Cespedes R., Gattino F., Biolatti B., Sasaki N., Nakagawa T., Di Renzo M.F. (2013). Activation of mammalian target of rapamycin (mTOR) in triple negative feline mammary carcinomas. BMC Vet. Res..

[56] Murakami Y., Tateyama S., Rungsipipat A., Uchida K., Yamaguchi R. (2000). Immunohistochemical Analysis of Cyclin A, Cyclin D1 and P53 in Mammary Tumors, Squamous Cell Carcinomas and Basal Cell Tumors of Dogs and Cats. J. Vet. Med. Sci..

[57] Nakano M., Wu H., Taura Y., Inoue M. (2006). Immunohistochemical detection of Mdm2 and p53 in feline mammary gland tumors. J. Vet. Med. Sci..

[58] Morris J.S., Nixon C., Bruck A., Nasir L., Morgan I.M., Philbey A.W. (2008). Immunohistochemical expression of TopBP1 in feline mammary neoplasia in relation to histological grade, Ki67, ERα and p53. Vet. J..

[59] Müller A., Homey B., Soto H., Ge N., Catron D., Buchanan M.E., McClanahan T., Murphy E., Yuan W., Wagner S.N. (2001). Involvement of chemokine receptors in breast cancer metastasis. Nature.

[60] Marques C.S., Santos A.R., Gameiro A., Correia J., Ferreira F. (2018). CXCR4 and its ligand CXCL12 display opposite expression profiles in feline mammary metastatic disease, with the exception of HER2-overexpressing tumors. BMC Cancer.

[61] Liu F., Lang R., Wei J., Fan Y., Cui L., Gu F., Guo X., Pringle G.A., Zhang X., Fu L. (2009). Increased expression of SDF-1/CXCR4 is associated with lymph node metastasis of invasive micropapillary carcinoma of the breast. Histopathology.

[62] Marques C.S., Soares M., Santos A., Correia J., Ferreira F. (2017). Serum SDF-1 levels are a reliable diagnostic marker of feline mammary carcinoma, discriminating HER2-overexpressing tumors from other subtypes. Oncotarget.

[63] Nascimento C., Gameiro A., Ferreira J., Correia J., Ferreira F. (2021). Diagnostic Value of VEGF-A, VEGFR-1 and VEGFR-2 in Feline Mammary Carcinoma. Cancers.

[64] Nascimento C., Urbano A.C., Gameiro A., Correia J., Ferreira F. (2020). Serum PD-1/PD-L1 Levels, Tumor Expression and PD-L1 Somatic Mutations in HER2-Positive and Triple Negative Normal-Like Feline Mammary Carcinoma Subtypes. Cancers.

[65] Papadaki M.A., Koutsopoulos A.V., Tsoulfas P.G., Lagoudaki E., Aggouraki D., Monastirioti A., Koutoulaki C., Apostolopoulou C.A., Merodoulaki A.C., Papadaki C. (2020). Clinical relevance of immune checkpoints on circulating tumor cells in breast cancer. Cancers.

[66] Gameiro A., Nascimento C., Urbano A.C., Correia J., Ferreira F. (2021). Serum levels and tumour expression of leptin and leptin receptor as promising clinical biomarkers of specific feline mammary carcinoma subtypes. Front. Vet. Sci..

[67] Hosney M., Sabet S., Shinawi M.E.L. (2017). Leptin is overexpressed in the tumor microenvironment of obese patients with estrogen receptor positive breast cancer. Exp. Ther. Med..

[68] Pan H., Deng L.-L., Cui J.-Q., Shi L., Yang Y.-C., Luo J.-H., Qin D., Wang L. (2018). Association between serum leptin levels and breast cancer risk: An updated systematic review and meta-analysis. Medicine.

[69] Shibata H., Sasaki N., Honjoh T., Ohishi I., Takiguchi M., Ishioka K., Ahmed M., Soliman M., Kimura K., Saito M. (2003). Feline Leptin: Immunogenic and Biological Activities of the Recombinant Protein, and Its Measurement by ELISA. J. Vet. Med. Sci..

[70] Georgiou G.P., Provatopoulou X., Kalogera E., Siasos G., Menenakos E., Zografos G.C., Gounaris A. (2016). Serum resistin is inversely related to breast cancer risk in premenopausal women. Breast.

[71] Saxena N., Taliaferro-Smith L., Knight B.B., Merlin D., Anania F.A., O’Regan R.M., Sharma D. (2008). Bidirectional Crosstalk between Leptin and Insulin-Like Growth Factor-1 Signaling Promotes Invasion and Migration of Breast Cancer Cells via Transactivation of Epidermal Growth Factor Receptor. Cancer Res..

[72] Liang X., Wang S., Wang X., Zhang L., Zhao H., Zhang L. (2018). Leptin promotes the growth of breast cancer by upregulating the wnt/β-catenin pathway. Exp. Ther. Med..

[73] Martín-Romero C., Santos-Alvarez J., Goberna R., Sánchez-Margalet V. (2000). Human leptin enhances activation and proliferation of human circulating T lymphocytes. Cell. Immunol..

[74] Kim S.Y., Lim J.H., Choi S.W., Kim M., Kim S.T., Kim M.S., Cho Y.S., Chun E., Lee K.Y. (2010). Preferential effects of leptin on CD4 T cells in central and peripheral immune system are critically linked to the expression of leptin receptor. Biochem. Biophys. Res. Commun..

[75] Miyoshi Y., Funahashi T., Tanaka S., Taguchi T., Tamaki Y., Shimomura I., Noguchi S. (2006). High expression of leptin receptor mRNA in breast cancer tissue predicts poor prognosis for patients with high, but not low, serum leptin levels. Int. J. Cancer.

[76] Ishikawa M., Kitayama J., Nagawa H. (2004). Enhanced expression of leptin and leptin receptor (OB-R) in human breast cancer. Clin. Cancer Res..

[77] Urbano A.C., Nascimento C., Soares M., Correia J., Ferreira F. (2020). Clinical Relevance of the serum CTLA-4 in Cats with Mammary Carcinoma. Sci. Rep..

[78] Li S., Chen L., Jiang J. (2019). Role of programmed cell death ligand-1 expression on prognostic and overall survival of breast cancer: A systematic review and meta-analysis. Medicine.

[79] Li Y., Cui X., Yang Y.J., Chen Q.Q., Zhong L., Zhang T., Cai R.L., Miao J.Y., Yu S.C., Zhang F. (2019). Serum sPD-1 and sPD-L1 as Biomarkers for Evaluating the Efficacy of Neoadjuvant Chemotherapy in Triple-Negative Breast Cancer Patients. Clin. Breast Cancer.

[80] Michishita M., Ohtsuka A., Nakahira R., Tajima T., Nakagawa T., Sasaki N., Arai T., Takahashi K. (2016). Anti-tumor effect of bevacizumab on a xenograft model of feline mammary carcinoma. J. Vet. Med. Sci..

[81] Jeglum K.A., DeGuzman E., Young K.M. (1985). Chemotherapy of advanced mammary adenocarcinoma in 14 cats. J. Am. Vet. Med. Assoc..

[82] Uyama R., Hong S.-H., Nakagawa T., Yazawa M., Kadosawa T., Mochizuki M., Tsujimoto H., Nishumura R., Sasaki N. (2005). Establishment and Characterization of Eight Feline Mammary Adenocarcinoma Cell Lines. J. Vet. Med. Sci..

[83] Chuang H., Lin Y., Chen T. (2021). Establishment and Characterization of a Feline Mammary Tumor Patient-Derived Xenograft Model Methods. BMC Vet. Res..

[84] Hassan B.B., Elshafae S.M., Supsavhad W., Simmons J.K., Dirksen W.P., Sokkar S.M., Rosol T.J. (2017). Feline Mammary Cancer: Novel Nude Mouse Model and Molecular Characterization of Invasion and Metastasis Genes. Vet. Pathol..

[85] Figueira A.C., Gomes C., Mendes N., Amorim I., de Matos A.J.F., Dias-Pereira P., Gärtner F. (2016). Catenin Adhesion Complex in a Feline Mammary Carcinoma Cell Line. Clin. Diagn. Pathol..

[86] Subik K., Lee J.-F., Baxter L., Strzepek T., Costello D., Crowley P., Xing L., Hung M.-C., Bonfiglio T., Hicks D.G. (2010). The Expression Patterns of ER, PR, HER2, CK5/6, EGFR, Ki-67 and AR by Immunohistochemical Analysis in Breast Cancer Cell Lines. Breast Cancer.

[87] Rangarajan A., Weinberg R.A. (2003). Comparative biology of mouse versus human cells: Modelling human cancer in mice. Nat. Rev. Cancer.

[88] De Las Mulas J.M., Reymundo C. (2000). Animal models of human breast carcinoma: Canine and feline neoplasms. Clin. Transl. Oncol..

[89] Yamashita-Kashima Y., Shu S., Yorozu K., Moriya Y., Harada N. (2017). Mode of action of pertuzumab in combination with trastuzumab plus docetaxel therapy in a HER2-positive breast cancer xenograft model. Oncol. Lett..

[90] Bonkobara M. (2015). Dysregulation of tyrosine kinases and use of imatinib in small animal practice. Vet. J..

[91] Agus D.B., Akita R.W., Fox W.D., Lewis G.D., Higgins B., Pisacane P.I., Lofgren J.A., Tindell C., Evans D.P., Maiese K. (2002). Targeting ligand-activated ErbB2 signaling inhibits breast and prostate tumor growth. Cancer Cell.

[92] Scheuer W., Friess T., Burtscher H., Bossenmaier B., Endl J., Hasmann M. (2009). Strongly enhanced antitumor activity of trastuzumab and pertuzumab combination treatment on HER2-positive human xenograft tumor models. Cancer Res..

[93] Baselga J., Gelmon K.A., Verma S., Wardley A., Conte P.F., Miles D., Bianchi G., Cortes J., McNally V.A., Ross G.A. (2010). Phase II trial of pertuzumab and trastuzumab in patients with human epidermal growth factor receptor 2-positive metastatic breast cancer that progressed during prior trastuzumab therapy. J. Clin. Oncol..

[94] Metzger-Filho O., Winer E.P., Krop I. (2013). Pertuzumab: Optimizing HER2 blockade. Clin. Cancer Res..

[95] Klapper L.N., Waterman H., Sela M., Yarden Y. (2000). Tumor-inhibitory antibodies to HER-2/ErbB-2 may act by recruiting c-Cbl and enhancing ubiquitination of HER-2. Cancer Res..

[96] Piccart-Gebhart M.J., Procter M., Leyland-Jones B., Goldhirsch A., Untch M., Smith I., Gianni L., Baselga J., Bell R., Jackisch C. (2005). Trastuzumab after Adjuvant Chemotherapy in HER2-Positive Breast Cancer. N. Engl. J. Med..

[97] Nahta R., Yuan L.X.H., Du Y., Esteva F.J. (2007). Lapatinib induces apoptosis in trastuzumab-resistant breast cancer cells: Effects on insulin-like growth factor I signaling. Mol. Cancer Ther..

[98] Slamon D., Eiermann W., Robert N., Pienkowski T., Martin M., Press M., Mackey J., Glaspy J., Chan A., Pawlicki M. (2011). Adjuvant Trastuzumab in HER2-Positive Breast Cancer. N. Engl. J. Med..

[99] Menyhart O., Santarpia L., Gyorffy B. (2015). A Comprehensive Outline of Trastuzumab Resistance Biomarkers in HER2 Overexpressing Breast Cancer. Curr. Cancer Drug Targets.

[100] Kast K., Schoffer O., Link T., Forberger A., Petzold A., Niedostatek A., Werner C., Klug S.J., Werner A., Gatzweiler A. (2017). Trastuzumab and survival of patients with metastatic breast cancer. Arch. Gynecol. Obstet..

[101] Lambert J.M., Chari R.V.J. (2014). Ado-trastuzumab emtansine (T-DM1): An antibody-drug conjugate (ADC) for HER2-positive breast cancer. J. Med. Chem..

[102] Lacasse V., Beaudoin S., Jean S., Leyton J.V. (2020). A Novel Proteomic Method Reveals NLS Tagging of T-DM1 Contravenes Classical Nuclear Transport in a Model of HER2-Positive Breast Cancer. Mol. Ther. Methods Clin. Dev..

[103] Liu P., Fan J., Wang Z., Zai W., Song P., Li Y., Ju D. (2020). The role of autophagy in the cytotoxicity induced by trastuzumab emtansine (T-DM1) in HER2-positive breast cancer cells. AMB Express.

[104] Franklin M.C., Carey K.D., Vajdos F.F., Leahy D.J., De Vos A.M., Sliwkowski M.X. (2004). Insights into ErbB signaling from the structure of the ErbB2-pertuzumab complex. Cancer Cell.

[105] Diermeier-Daucher S., Breindl S., Buchholz S., Ortmann O., Brockhoff G. (2011). Modular anti-EGFR and anti-Her2 targeting of SK-BR-3 and BT474 breast cancer cell lines in the presence of ErbB receptor-specific growth factors. Cytom. Part A.

[106] Li J., Ma M., Yang X., Zhang M., Luo J., Zhou H., Huang N., Xiao F., Lai B., Lv W. (2020). Circular HER2 RNA positive triple negative breast cancer is sensitive to Pertuzumab. Mol. Cancer.

[107] Sakai K., Yokote H., Murakami-murofushi K., Tamura T., Saijo N., Nishio K. (2007). Pertuzumab, a novel HER dimerization inhibitor, inhibits the growth of human lung cancer cells mediated by the HER3 signaling pathway. Cancer Sci..

[108] Burguin A., Furrer D., Ouellette G., Jacob S., Diorio C., Durocher F. (2020). Trastuzumab effects depend on HER2 phosphorylation in HER2-negative breast cancer cell lines. PLoS ONE.

[109] Endo Y., Takeda K., Mohan N., Shen Y., Jiang J., Rotstein D., Wu W.J. (2018). Payload of T-DM1 binds to cell surface cytoskeleton-associated protein 5 to mediate cytotoxicity of hepatocytes. Oncotarget.

[110] Weigelt B., Lo A.T., Park C.C., Gray J.W., Bissell M.J. (2010). HER2 signaling pathway activation and response of breast cancer cells to HER2-targeting agents is dependent strongly on the 3D microenvironment. Breast Cancer Res. Treat..

[111] Tatara T., Mukohara T., Tanaka R., Shimono Y., Funakoshi Y., Imamura Y., Toyoda M., Kiyota N., Hirai M., Kakeji Y. (2018). 3D Culture Represents Apoptosis Induced by Trastuzumab Better than 2D Monolayer Culture. Anticancer. Res..

[112] Matkar S., An C., Hua X. (2017). Kinase inhibitors of HER2/AKT pathway induce ERK phosphorylation via a FOXO-dependent feedback loop. Am. J. Cancer Res..

[113] Faber A.C., Li D., Song Y.C., Liang M.C., Yeap B.Y., Bronson R.T., Lifshits E., Chen Z., Maira S.M., García-Echeverría C. (2009). Differential induction of apoptosis in HER2 and EGFR addicted cancers following PI3K inhibition. Proc. Natl. Acad. Sci. USA.

[114] O’Brien N.A., Browne B.C., Chow L., Wang Y., Ginther C., Arboleda J., Duffy M.J., Crown J., O’Donovan N., Slamon D.J. (2010). Activated phosphoinositide 3-kinase/AKT signaling confers resistance to trastuzumab but not lapatinib. Mol. Cancer Ther..

[115] Frenel J.S., Bourbouloux E., Berton-Rigaud D., Sadot-Lebouvier S., Zanetti A., Campone M. (2009). Lapatinib in metastatic breast cancer. Women’s Heal..

[116] Opdam F.L., Guchelaar H., Beijnen J.H., Schellens J.H.M. (2012). Lapatinib for Advanced or Metastatic Breast Cancer. Oncologist.

[117] Shi H., Zhang W., Zhi Q., Jiang M. (2016). Lapatinib resistance in HER2+ cancers: Latest findings and new concepts on molecular mechanisms. Tumor Biol..

[118] Stanley A., Ashrafi G.H., Seddon A.M., Modjtahedi H. (2017). Synergistic effects of various Her inhibitors in combination with IGF-1R, C-MET and Src targeting agents in breast cancer cell lines. Sci. Rep..

[119] Tiwari S.R., Mishra P., Abraham J. (2015). Neratinib, A Novel HER2-Targeted Tyrosine Kinase Inhibitor. Clin. Breast Cancer.

[120] Carmona F.J., Montemurro F., Kannan S., Rossi V., Verma C., Baselga J., Scaltriti M. (2017). AKT signaling in ERBB2-amplified breast cancer F. Physiol. Behav..

[121] Kidger A.M., Sipthorp J., Cook S.J. (2018). ERK1/2 inhibitors: New weapons to inhibit the RAS-regulated RAF-MEK1/2-ERK1/2 pathway. Pharmacol. Ther..

[122] Nielsen T.O., Hsu F.D., Jensen K., Cheang M., Karaca G., Hu Z., Hernandez-Boussard T., Livasy C., Cowan D., Dressler L. (2004). Immunohistochemical and clinical characterization of the basal-like subtype of invasive breast carcinoma. Clin. Cancer Res..

[123] Song X., Liu Z., Yu Z. (2020). EGFR promotes the development of triple negative breast cancer through JAK/STAT3 signaling. Cancer Manag. Res..

[124] Chen Y.J., Yeh M.H., Yu M.C., Wei Y.L., Chen W.S., Chen J.Y., Shih C.Y., Tu C.Y., Chen C.H., Hsia T.C. (2013). Lapatinib-induced NF-kappaB activation sensitizes triple-negative breast cancer cells to proteasome inhibitors. Breast Cancer Res..

[125] Liu C.Y., Hu M.H., Hsu C.J., Huang C.T., Wang D.S., Tsai W.C., Chen Y.T., Lee C.H., Chu P.Y., Hsu C.C. (2016). Lapatinib inhibits CIP2A/PP2A/p-Akt signaling and induces apoptosis in triple negative breast cancer cells. Oncotarget.

[126] Scaltriti M., Verma C., Guzman M., Jimenez J., Parra J.L., Pedersen K., Landolfi S., Ramon y Cajal S., Arribas J., Baselga J. (2009). Lapatinib, a HER2 tyrosine kinase inhibitor, induces stabilization and accumulation of HER2 and potentiates trastuzumab-dependent cell cytotoxicity. Oncogene.

[127] McGowan P.M., Mullooly M., Caiazza F., Sukor S., Madden S.F., Maguire A.A., Pierce A., McDermott E.W., Crown J., O’Donovan N. (2013). ADAM-17: A novel therapeutic target for triple negative breast cancer. Ann. Oncol..

[128] Nagpal A., Redvers R.P., Ling X., Ayton S., Fuentes M., Tavancheh E., Diala I., Lalani A., Loi S., David S. (2019). Neoadjuvant neratinib promotes ferroptosis and inhibits brain metastasis in a novel syngeneic model of spontaneous HER2+ve breast cancer metastasis. Breast Cancer Res..

[129] Breslin S., Lowry M.C., O’Driscoll L. (2017). Neratinib resistance and cross-resistance to other HER2-targeted drugs due to increased activity of metabolism enzyme cytochrome P4503A4. Br. J. Cancer.

[130] Rani S., Corcoran C., Shiels L., Germano S., Breslin S., Madden S., McDermott M.S., Browne B.C., O’Donovan N., Crown J. (2014). Neuromedin U: A candidate biomarker and therapeutic target to predict and overcome resistance to HER-tyrosine kinase inhibitors. Cancer Res..

[131] Tóth G., Szöőr Á., Simon L., Yarden Y., Szöllősi J., Vereb G. (2016). The combination of trastuzumab and pertuzumab administered at approved doses may delay development of trastuzumab resistance by additively enhancing antibody-dependent cell-mediated cytotoxicity. MAbs.

[132] Nahta R., Hung M.C., Esteva F.J. (2004). The HER-2-Targeting Antibodies Trastuzumab and Pertuzumab Synergistically Inhibit the Survival of Breast Cancer Cells. Cancer Res..

[133] Okita R., Shimizu K., Nojima Y., Yukawa T., Maeda A., Saisho S., Nakata M. (2015). Lapatinib enhances trastuzumab-mediated antibody-dependent cellular cytotoxicity via upregulation of HER2 in malignant mesothelioma cells. Oncol. Rep..

[134] Watson S.S., Dane M., Chin K., Tatarova Z., Liu M., Liby T., Thompson W., Smith R., Nederlof M., Bucher E. (2018). Microenvironment-Mediated Mechanisms of Resistance to HER2 Inhibitors Differ between HER2+ Breast Cancer Subtypes. Cell Syst..

[135] Canonici A., Ivers L., Conlon N.T., Pedersen K., Gaynor N., Browne B.C., O’Brien N.A., Gullo G., Collins D.M., O’Donovan N. (2019). HER-targeted tyrosine kinase inhibitors enhance response to trastuzumab and pertuzumab in HER2-positive breast cancer. Investig. New Drugs.

[136] Liu T., Yacoub R., Taliaferro-Smith L.D., Sun S.-Y., Graham T.R., Dolan R., Lobo C., Tighiouart M., Yang L., Adams A. (2011). Combinatorial Effects of Lapatinib and Rapamycin in Triple-Negative Breast Cancer Cells. Mol. Cancer Ther..

[137] Mallon R., Feldberg L.R., Lucas J., Chaudhary I., Dehnhardt C., Delos Santos E., Chen Z., Dos Santos O., Ayral-Kaloustian S., Venkatesan A. (2011). Antitumor efficacy of PKI-587, a highly potent dual PI3K/mTOR kinase inhibitor. Clin. Cancer Res..

[138] Ogawa L., Lindquist D. (2018). Dual HER2 Suppression with Lapatinib plus Trastuzumab for Metastatic Inflammatory Breast Cancer: A Case Report of Prolonged Stable Disease. Case Rep. Oncol..

[139] Noh W., Mondesire W.H., Peng J., Jian W., Zhang H. (2004). Determinants of Rapamycin Sensitivity in Breast Cancer Cells. Clin. Cancer Res..

[140] Jhanwar-Uniyal M., Wainwright J.V., Mohan A.L., Tobias M.E., Murali R., Gandhi C.D., Schmidt M.H. (2019). Diverse signaling mechanisms of mTOR complexes: mTORC1 and mTORC2 in forming a formidable relationship. Adv. Biol. Regul..

[141] Walsh S., Flanagan L., Quinn C., Evoy D., McDermott E.W., Pierce A., Duffy M.J. (2012). MTOR in breast cancer: Differential expression in triple-negative and non-triple-negative tumors. Breast.

[142] Crown J., O’Shaughnessy J., Gullo G. (2012). Emerging targeted therapies in triple-negative breast cancer. Ann. Oncol..

[143] Gandhi L., Bahleda R., Tolaney S.M., Kwak E.L., Cleary J.M., Pandya S.S., Hollebecque A., Abbas R., Ananthakrishnan R., Berkenblit A. (2014). Phase i study of neratinib in combination with temsirolimus in patients with human epidermal growth factor receptor 2-dependent and other solid tumors. J. Clin. Oncol..

[144] Mcdonnel S.J., Tell L.A., Murphy B.G. (2013). Pharmacokinetics and pharmacodynamics of suberoylanilide hydroxamic acid in cats. J. Vet. Pharmacol. Ther..

[145] Samantha J.M., Liepnieks M.L., Murphy B.G. (2014). Treatment of chronically FIV-infected cats with suberoylanilide hydroxamic acid. Antivir. Res..

[146] Ediriweera M.K., Tennekoon K.H., Samarakoon S.R. (2019). Emerging role of histone deacetylase inhibitors as anti-breast-cancer agents. Drug Discov. Today.

[147] Villanueva C.B., Bazan F.F., Pivot X.B. (2013). New microtubule inhibitors in breast cancer. Curr. Breast Cancer Rep..

[148] Xu W.S., Parmigiani R.B., Marks P.A. (2007). Histone deacetylase inhibitors: Molecular mechanisms of action. Oncogene.

[149] Chun P. (2015). Histone deacetylase inhibitors in hematological malignancies and solid tumors. Arch. Pharm. Res..

[150] Kamarulzaman N.S., Dewadas H.D., Leow C.Y., Yaacob N.S., Mokhtar N.F. (2017). The role of REST and HDAC2 in epigenetic dysregulation of Nav1.5 and nNav1.5 expression in breast cancer. Cancer Cell Int..

[151] Cui Z., Xie M., Wu Z., Shi Y. (2018). Relationship between histone deacetylase 3 (HDAC3) and breast cancer. Med. Sci. Monit..

[152] Yamashita Y.I., Shimada M., Harimoto N., Rikimaru T., Shirabe K., Tanaka S., Sugimachi K. (2003). Histone deacetylase inhibitor trichostatin a induces cell-cycle arrest/apoptosis and hepatocyte differentiation in human hepatoma cells. Int. J. Cancer.

[153] Rene Gonzalez R., Watters A., Xu Y., Singh U.P., Mann D.R., Rueda B.R., Penichet M.L. (2009). Leptin-signaling inhibition results in efficient anti-tumor activity in estrogen receptor positive or negative breast cancer. Breast Cancer Res..

[154] Cimino-Mathews A., Thompson E., Taube J.M., Ye X., Lu Y., Meeker A., Xu H., Sharma R., Lecksell K., Cornish T.C. (2016). PD-L1 (B7-H1) expression and the immune tumor microenvironment in primary and metastatic breast carcinomas. Hum. Pathol..

[155] Sun Z., Lan X., Xu S., Li S., Xi Y. (2020). Efficacy of bevacizumab combined with chemotherapy in the treatment of HER2-negative metastatic breast cancer: A network meta-analysis. BMC Cancer.

